# Hpb News

**DOI:** 10.1155/1991/71341

**Published:** 1991


					HPB Surgery, 1991, Vol. 3, pp. 301-302
Reprints available directly from the publisher
Photocopying permitted by license only
(C) 1991 Harwood Academic Publishers GmbH
Printed in the United Kingdom
HPB NEWS
WORLD ASSOCIATION OF HPB SURGERY
FOURTH WORLD CONGRESS, HONG KONG
Plans are now well advanced for the next World Congress, which is being organised
by Professor Arthur Li and his colleagues. The Congress will be held from 7-11
June 1992 in Hong Kong. Details should be available by the end of February.
The final announcement and call for abstracts for Hong Kong is planned to be
circulated in July 1991, with a provisional closing date for receipt of abstracts by
30th November 1991. The Local Organising Committee and Scientific Committee
are conspiring to make this a very successful conference.
THE PROPOSED MERGER WITH IHBPA
Negotiations between this Association and the International Hepato-Biliary-
Pancreatic Association are proceeding very well. At the General Assembly
(Business Meeting) in London in June 1990 it was "decided that every effor.t
should be made to have a close co-operation or merger between the two
associations" (extract from the minutes). In August 1990 four representatives of
WAHPBS (Niels van der Heyde, Martin Adson, Kenneth Hobbs and R.C.N.
Williamson) met eight representatives of IHBPA in Paris. The meeting was
friendly and constructive. It was agreed that the aim should be complete integration
or merger between the two associations as soon as practicable. It was proposed that
the 1994 meeting should be held in Boston, USA, and that this should be a joint
meeting with one common social and scientific programme. Assuming that the
proposals are acceptable to both the existing associations, they will unite formally
in Boston to form one society with one membership and one set of officers. All
existing Members of WAHPBS and IHBPA will automatically become Members of
the new combined society, which (it is proposed) will then be known as the
International Hepato-Pancreato-Biliary Association.
ASIAN DEVELOPMENTS IN HPB SURGERY
The first Asian Conference took place recently (10-12 January 1991) in Bangkok,
Thailand. it was organised jointly by Dr. Tadahiro Takada (Japan) and Dr.
Thamnoon Varlyapongs (Thailand) with a strong supporting committee from each
of these countries. It was an enormous and very successful meeting with about 1000
participants. The volume of high quality papers, posters and videos underlined the
301
302 HPB NEWS
interest and experience of HPB surgeons throughout the continent of Asia. The
Asian Society of Hepato-Biliary-Pancreatic Surgery was founded at the conference,
with Dr. Takada as the first President. The new Society will hold two-yearly
meetings and will next meet in Taiwan in 1993; it is hoped that many of its Members
will attend the Hong Kong meeting in 1992. Every continent of the world has its
own spectrum of HPB disease, and there is much to learn from each other.
FORTHCOMING MEETINGS
Second National Conference of the Italian Chapter of the WAHPBS. Taormina,
Sicily, Italy. 9-11 June 1991 (please note change of dates).
Details from
Professor Gaetano Romeo
Istituto di Patologia Chirurgica II
Universita da Gatania
USL35 -Ospedale Vittorio Emanuele
Via Plebisolto, 624
95124 Catania
Italy.
First Alps-Adria Congress on Hepato-Pancreato-Biliary Surgery and Medicine.
Ljubljana, Yugoslavia 3-5 October 1991.
Organiser Professor Vladislav Pegan
Details from
Cankarjev Dom
Cultural and Congress Centre
Congress Department
Kidricev Park 1
6100 Ljubljana
Slovenija Yugoslavia
Closing date for abstracts: 15 March 1991.

				

